# Pollution and Health Risk Assessments of Potentially Toxic Elements in Soil and Sediment Samples in a Petrochemical Industry and Surrounding Area

**DOI:** 10.3390/molecules24112139

**Published:** 2019-06-06

**Authors:** Dubravka Relić, Sanja Sakan, Ivan Anđelković, Aleksandar Popović, Dragana Đorđević

**Affiliations:** 1 Faculty of Chemistry, University of Belgrade, Studentski trg 12-16, 11000 Belgrade, Serbia; apopovic@chem.bg.ac.rs; 2 Centre of Excellence in Environmental Chemistry and Engineering–ICTM, University of Belgrade, Njegoševa 12, 11158 Belgrade, Serbia; ssakan@chem.bg.ac.rs; 3 Innovation Center, Faculty of Chemistry, University of Belgrade, Studentski trg 12-16, 11000 Belgrade, Serbia; ivan.andelkovic@adelaide.edu.au

**Keywords:** potentially toxic elements, petrochemical industry-workers, non-industrial area-residential people, pollution indices, health risk assessment

## Abstract

The pollution state and health risk assessment of potentially toxic elements (PTE) in soil and sediment samples of the petrochemical industry and its surrounding area are evaluated in this study. The pseudo-total contents of Ba, Cd, Co, Cu, Cr, Mn, Ni, Pb, V, Zn, As, Hg, and Se were measured by inductively coupled plasma–optical emission spectrometry (ICP/OES) in analyzed samples. Instead of determining total content, we performed aqua regia of the samples. The silicate matrix remained, and the quantities of elements that are within the silicate matrix do not represent an environmental danger. The soils from the chlor–alkali plant are highly polluted by Hg (the enrichment factor values were above 6000), and by Cu, Cd, Pb, and Zn, while the sediment samples from the wastewater channel are polluted with Cr, Cd, and Hg. The measured element contents are used for calculating health risk criteria for a composite worker (a worker who is exposed, long-term, during the work day) and for residential people. Hg is the element that mainly contributes to non-carcinogenic risks within the petrochemical area. The highest value of total carcinogenic risk obtained in the sediment sample from the wastewater channel, and the metal that mostly contributes is Cr. The areas closest to the petrochemical industry have higher values of health risk criteria parameters and pollution indices. The areas that are located further to the north and south from the petrochemical industry are less burdened with the analyzed elements, which is significant because the closest city and village are situated in those directions.

## 1. Introduction

Potentially toxic elements are some of the most ubiquitous pollutants today. The significant threat that comes from these elements is accumulation in the environment since they are non-biodegradable [1], and two main origins of trace elements, and potentially toxic elements, in soil and sediment samples are natural and anthropogenic. The weathering of rocks is one of the natural sources while the anthropogenic elements have diverse human influences, including chemical and petrochemical industries, as well as social activities such as transportation and agriculture [2,3,4]. With the increase of industrialization and urbanization, the pollution by elements will increase [5,6] and the exposure to them can lead to acute and chronic toxicity [7]. For assessing soil and sediment contamination, researchers recommended the calculation of pollution indices [1,4,8,9,10,11]. Besides being a significant pollutant of soil and sediments, potentially toxic elements (PTE) can also pose a substantial threat to human health. Humans can be exposed to contamination from soil and sediments by different routes, such as ingestion, inhalation of soil particles, and dermal contact. For obtaining the health risk assessment, two indexes can be calculated: non-carcinogenic and carcinogenic risks, in the form of the hazard quotient (HQ) and cancer risk, respectively.

Some of the analyzed PTE, such as Co, Cr, Cu, Mn, Ni, Se, and Zn are essential to biological systems and can be harmful if present in excess. On the other hand, the rest of the analyzed elements are detrimental to humans, animals, and crops. If PTE is present in soil due to anthropogenic activities, they can disturb the normal functioning of soil biota and the entire soil system. Based on the toxicological experiments, As, Cr, and Pb are known to be human carcinogens; Cd, Ni, and Co are also probable human carcinogens through an inhalation route. Besides the carcinogenic threat, these and the rest of the analyzed PTEs have some toxical, non-carcinogenic effects on human health.

The first aspect of this work is to evaluate pollution indices by calculating the contamination factors, such as enrichment factor (EnF), pollution load index (PLI), total enrichment factor (R), and ecological risk index (ERI), to estimate the anthropogenic input of the elements and to assess the pollution state of the investigated area. Secondly, we compared the pseudo-total concentration of PTE with a threshold effect concentration (TEC) and a probable effect concentration (PEC) that were presented in a paper by MacDonald et al. [12] as obtaining adverse biological effects. Thirdly, to achieve the health risk criteria, the hazard quotient (HQ) and cancer risk were calculated. Two scenarios are taken into acount—the health risk criteria for the adults who can be potentially exposed to the soil during occupational activities at the site as composite workers, and the residential people. Besides the samples from the petrochemical industry, we analyzed the samples that are around this industry (residential areas), so the scenario of residential soil exposure was also applied. Pančevo city and Starčevo village lie in the vicinity of this industry. Moreover, the scenario for health risk criteria for adjustable residential exposure (i.e., adjustable by considering the different age groups, children and adults), were calculated. After obtaining these results, the PCA (principal component analysis) and correlation analysis were used to see how the contamination factors are in associatios with the health risk criteria for obtaining an overall picture of the pollution state of the analyzed area.

## 2. Results and Discussion

In Table 1, some basic statistics of elements contents are presented, with the percentage of the pseudo-total element concentration of the analyzed samples that are above the threshold and probable effect contents. The basic statistics of the elements calculated according to their pseudo-total contents presented in Table S1 (Supplementary Material).

According to the coefficient of variation, Ba, Co, Mn, Ni, V, and As have a uniform distribution among both sets of samples, industrial and non-industrial, since the coefficients of variation are less than 100%. For the rest of the elements, this is not the case.

Coefficients of variation above 100% have Cr, Cu, Hg, Cd, Pb, Zn, and Se in samples within the industrial area, indicating non-uniform distributions in the analyzed samples. The average value of Hg (35.6 mg kg^−1^ ± 69.2 mg kg^−1^) in the petrochemical samples is more than ten times above the maximum allowable Hg content in Serbia (Table 1), while the maximum detected values of Cr, Cu, Ni, Pb, and Zn were significantly above their permissible concentrations. Elements that have higher average values in the non-industrial area are Co, Mn, Ni, V, and As. Those elements represent indicators of pollution by the petrochemical industry [13,14]. The obtained mean values for Pb, V, and Cr in the industrial samples are higher than those reported by Nadal et al. [15] for soil samples from Tarragona (Spain). The contents of As, Cd, Hg, and Mn are higher than those presented in Nadal et al. [15] (where the mean value for As was 5.5 mg kg^−1^, Cd was 0.21 mg kg^−1^, Hg was 0.08 mg kg^−1^, and Mn was 212.5 mg kg^−1^) and higher than in soils from Alcalá [16] (where the mean value for As was 4.83 mg kg^−1^; Cd was 0.11 mg kg^−1^; Hg was not detected and Mn was 99.27 mg kg^−1^). Compared with the values of elements determined in soils from the eastern part of Serbia (from Bor Copper Smelting Plant) the obtained Hg contents are higher than 0.133 mg kg ^−1^, while the obtained values of Cu, Pb, Cd, Ni, Mn, and As are lower than those presented in the work of Nikolić et al. [17] (the mean value for Cu was 913.33 mg kg^−1^, Pb was 86.67 mg kg^−1^, Cd was 2.92 mg kg^−1^, Ni was 36.83 mg kg^−1^, Mn was 460 mg kg^−1^ and As was 15 mg kg^−1^.

The most dangerous element in our investigation is Hg, since 76% of the samples within the industrial area have this toxic element in a concentration above PEC (Table 1). The higher values of TEC were obtained for non-industrial samples; in the case of Ni and Hg—for all the samples—their values are above the TEC (100%). Although the detected contents of Ni were mainly below the maximum allowable concentration and background values, when comparing PEC values, 44% of the samples from the non-industrial area have Ni in concentrations that could cause adverse biological effects. In the case of other elements, Cr, Cu, Pb, Zn, and Hg have a lower percentage of samples with higher PEC values in non-industrial samples. On the other side, the TEC values obtained from the non-industrial area are higher than those from the petrochemical samples, which could imply that this industry represents the pollution source for surroundings but not in quantities that could impose higher adverse biological effects than in industrial samples (Table 1).

**Figure 1:**
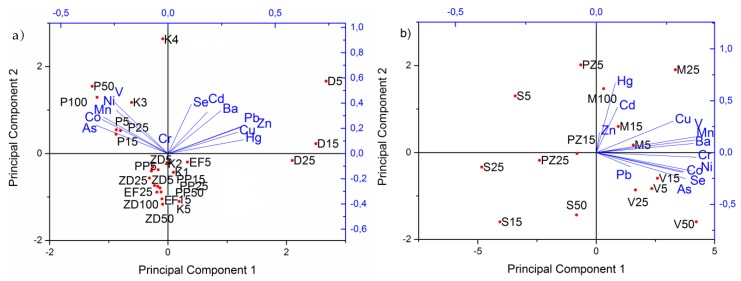
Principal component analysis (PCA) of extracted elements and samples within (**a**) industrial and (**b**) non-industrial areas.

Figure 1a,b show the loading plots of two significant principal components for both analyzed set of samples, i.e., PCA analysis showed the distribution of elements and samples from the industrial and non-industrial areas. In samples from the chlor–alkali plant (D), the dominant elements are Hg, Cu, Zn, Pb, Ba, Cd, and Se while V, Ni, Mn, Co, and As are present in higher concentrations in samples from the road in the petrochemical industry (P) and in the sediment K3 sample. The contents of Hg in the analyzed samples from the chlor–alkali plant (D) (Table S1) (203 mg kg^−1^; 237 mg kg^−1^; 162 mg kg^−1^; in D5, D15, and D15, respectively) are higher than those presented by Frentiu et al. [18] and by Le Faucheur et al. [19]. Similar contents of total Hg were identified in samples near a chemical plant in Quingzhen [20], in samples near a chlor–alkali plant in Nicarauga [21], and in samples from the former chlor–alkali plant [22]. The obtained Hg values are higher than the concentrations in sediments from the chlor–alkali complex in Iran [23].

Within the samples from non-industrial areas, it can be noted that the samples nearer to industry, Messer (M) and Vojlovica (V), showed the highest contents of elements than the samples from residential areas, Pančevo city (PZ) and Starčevo (S), with the exception of the surface sample from the city (PZ 5) (Figure 1b). Samples with elevated Hg contents are PZ5 and those from the Messer location (M25 and M100) (Table S1). The highest concentrations of As and Cr were detected in the Vojlovica samples (Figure 1b, Table S1). Besides these elements, Ni, Co, and Se showed high contents in the Vojlovica (V) samples, as well.

### 2.1. Results of Environmental Assessment

In Table 2, the average values with a standard deviation of the EnF (enrichment factor) for each sampling point are presented.

The element with the highest EnF values is Hg, with over 6000 in samples from the chlor–alkali plant (D). The EnF values of Hg in samples from the electrolysis factory (EF), the mercury disposal area (ZD), and the wastewater channel (K) are significantly higher than 50, which indicates that these samples showed an extremely severe enrichment of mercury pollution. These values of the enrichment factor for Hg were more elevated than those in the sample that was taken near to a chlor–alkali plant, as presented in the work of Bolaños-Álvarez et al. [8]. During a NATO bombing in 1999 [26], the analyzed Petrochemical complex was destroyed, and a significant amount of Hg polluted the environment. This investigation came 11 years after the bombing, and in the meantime, this area was under some remediation actions. Besides Hg, the sediments from the chlor–alkali plant are polluted with Cu, Cd, Pb, and Zn; their EnF values are above 50, which indicates extremely severe enrichments. Selenium showed severe enrichment in samples from the chlor–alkali plant. In the case of Cr, the mean EnF value is above 5 in sediments from the chlor–alkali plant, which indicates a moderately severe enrichment. The EnF value of Se is above 5 in samples from the wastewater channel, while Cd and Cr are in quantities that showed severe enrichment in samples from the wastewater channel.

According to the EnF values, elements that do not pose a threat to the environment are Ba, Co, Mn, Ni, V, and As (Table 2).

**Figure 2:**
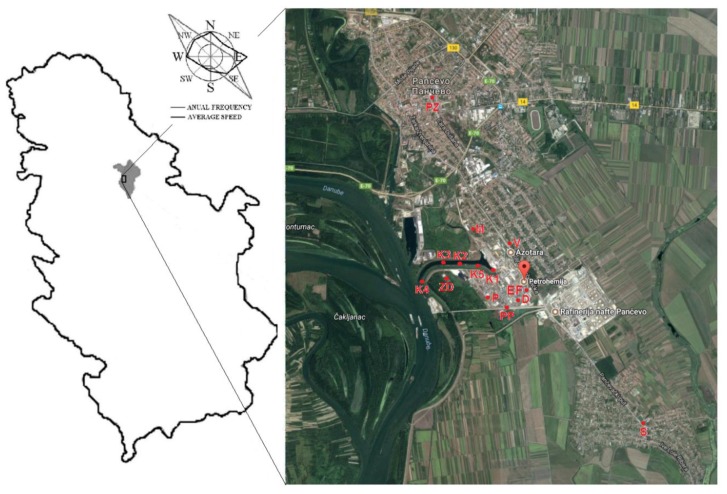
Location of the sampling points.

In Table 3, the mean values, with a standard deviation of the pollution indices, are presented, which we calculated according to the element contents shown in Table S1. The highest ecological risk index (ERI), above 600, has been calculated for samples from the industrial area (Table S2), D, EF, ZD, and K, whose ERI values indicate very high ecological risks, since the calculated mean values were 24,740, 7416, 1238, and 1307, respectively (Table 3). Further, the samples marked with P within the industrial area showed considerable ecological risk. Samples from the location marked with M and PZ (Figure 2), which are samples from the Messer location and Pančevo city, have ERI values slightly above 150 (Table 3). When we look carefully at Figure 2, it can be seen that these two locations are located northeastern from the petrochemical complex, and those samples showed a higher quantity of total Hg than the rest of the non-industrial samples (Figure 1b, Table S3). For the rest of the samples, the ERI values are below 150, which suggests that these samples have a low ecological risk, which is essential because some of the other analyzed samples are from urban and rural areas, located in the vicinity of a petrochemical complex.

The PLI showed little diversity in its values among the analyzed samples, similar to ERI. The values above 1 were present in samples of D and in samples from the wastewater channel (K). Greater diversity in the values was obtained by calculation of R, especially for samples of D, PE, ZD, and K (Table 3 (similar to ERI)) than for samples from the other sampling locations.

Elements that mainly contribute to elevated pollution indices are Hg, Cu, Pb, Zn, Cd, Se, and Cr in samples from the industrial area, while the samples from the non-industrial areas are not significantly polluted. Hg is the element that is the main pollutant, while its distribution to an area outside of the petrochemical complex is only evident in the locations that are northeasternly located from the industry complex (Messer and Pančevo city location) (Figure 1b).

According to pollution indices, the most polluted areas are those within the petrochemical complex: the chlor–alkali plant, the electrolysis factory, the mercury disposal area, and the wastewater channel.

### 2.2. Results of Health Risks Assessment

**Figure 3:**
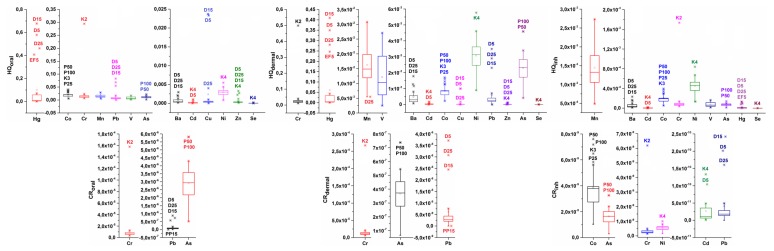
Hazard quotients (HQs) and cancer risk of elements for three exposure routes, oral, dermal, and inhalation (inh) in samples from the petrochemical complex.

**Figure 4:**
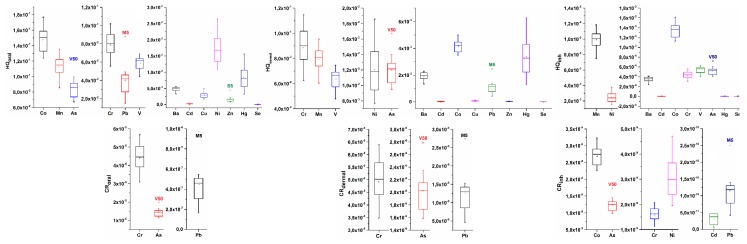
Hazard quotients (HQ) and cancer risk of elements for three exposure routes, oral, dermal, and inhalation (inh) in samples from the non-industrial area.

Besides the pollution indices, we calculated the human risk criteria for two exposure scenarios: (1) for the workers, and (2) for residential people. Box plots of three different exposure routes as HQs (oral, dermal, and inhalation) for each element were separately presented for samples from the industrial and the non-industrial areas (Figure 3 and Figure 4). The highest hazard quotient values were obtained from oral exposure, while the lowest were for inhalation. The samples that have the highest HQ value for oral non-carcinogenic risk (outliers in the box plots) are samples from the chlor–alkali plant (D) in the case of the Hg, Pb, Ba, Cu, Cd and Zn, wastewater channel (K), in the case of Cr, Co, Cd, Ni, Zn and Se, the road in the petrochemical industry (P) in the case of Co and As, and for the surface sample from the electrolysis facility (PE) in the case of Hg (Figure 3). The element that has the highest HQ_oral_ (oral hazard quotient) and HQ_dermal_ (dermal hazard quotient) values in all samples is from the chlor–alkali plant (D) and for the surface samples from the electrolysis facility (EF), the highest value is Hg. For the D15 sample (Figure 3), it is evident that the sum of HQ_oral_ and HQ_dermal_ is above 1 in the case of Hg, while this sum value is even higher when we add HQs of oral and dermal exposure for other elements. A sum value of HQ elements above 1 suggests a possible adverse non-carcinogenic health effect for workers. In sample K2, the highest HQoral value showed Cr, while Co and As were found in sediment samples from the road within the petrochemical industry (P) (Figure 3). The enrichment of Cr in sediments sampled at the K2 location can indicate the increment of Cr concentration because the main influent of wastewater from the Oil Refinery located is located in this location.

The elements that have the highest HQ_inh_ (inhale hazard quotient) are microwave digested from the samples in the industrial zone; these elements are Mn, Ni, and Co (Figure 3). Considering the HQs, for samples in the non-industrial area, the highest values are Co, Mn, and As for oral exposure, Cr, Mn, and V for dermal exposure, and, Mn and Ni, and slightly Co, as the threat for inhaling dust soil particles (Figure 4). Mn, Ni, and Co were also the elements that have the highest HQ_inh_ in samples from the industrial area, and their HQ_inh_ values from non-industrial areas are higher than those in industrial samples. Atmospheric transport could be one of the sources of pollution, and a possible hazard for the health of people from settlements, since housing settlements, both urban and rural, lie in the dominant wind direction (Figure 2). In the case of samples from urban and rural areas, Hg does not pose a threat, as the HQ this element is low, and the possible distribution of this toxic element from the petrochemical complex is not significant for the residential area. When we compare the HQs for a composite worker to those of residential people, we neglect the outliers; it can be noticed that slightly higher values in the residential area were obtained (Figure 3 and Figure 4). Authors in different scientific papers have shown that the hazard quotient is higher near the industrial area [15,27]. The atmospheric distribution and deposition of pollutants from industrial to residential areas could contribute to these slightly elevated HQ values. In residential areas, a significant contributor could also be diffuse pollution sources, such as traffic.

Three elements (Pb, Cr, and As) are involved in the calculation of carcinogenic risks for oral and dermal exposure, while for inhalation, the contents of Cd, Co, and Ni were also used. These elements have many industrial sources [13,14], and for the anthropogenic sources of soil, Pb can result from atmospheric deposition, mining, smelters, petrochemical industry, and vehicle emissions [28].

In both situations, the highest contribution to carcinogenic risk had Cr for all three exposure routes (Figure 3 and Figure 4). The maximal detected value was found in the K2 sample nearest to the effluent of the wastewater treatment plant from the Petrochemical Factory (Table S1), and the calculated carcinogenic risk was 4.98 × 10^−4^, as a sum of three exposure routes. This is a significant value for carcinogenic risk, since it is above the upper limit of the acceptable carcinogenic risk of 1·10^−4^. The average value for total carcinogenic risk within the industrial area is 4.50 × 10^−5^ ± 9.48 × 10^−5^ (Table S2), while within the non-industrial area, the average total carcinogenic risk is 1.09 × 10^−4^± 1.76 × 10^−5^ (Table S3). In this work, we calculated the worst-case scenario for the calculation of health risk assessment by using total Cr contents as Cr^6+^, which also elevated the value of carcinogenic risks. Besides Cr, As is an element that contributes to carcinogenic risk, especially in the case of non-industrial areas [29], because the highest values of this metalloid are detected in samples from non-industrial areas (Table 1), such as Vojlovica (V) (Figure 1b).

### 2.3. Correlation Analysis

**Figure 5:**
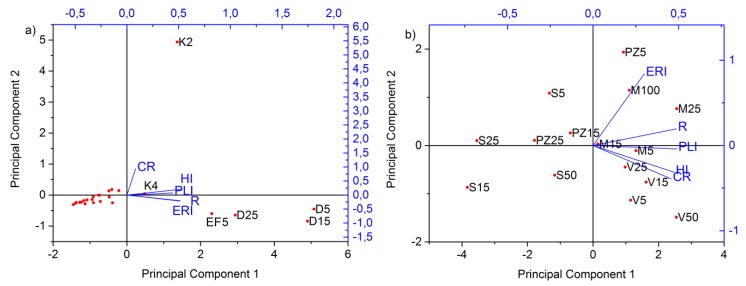
PCA analysis of pollution indices and health risk assessment in analyzed samples from (**a**) industrial and (**b**) non-industrial areas.

In Figure 5a,b, we presented PCA analysis of contamination indices and HRA parameters for the industrial and non-industrial samples. Within Table 4, Spearman correlation coefficients are shown alongside these variables. Good correlations were achieved when the pollution indices were correlated with non-carcinogenic risk, while there was just one significant correlation among cancer risk and PLI in samples from the petrochemical complex (Table 4a). The highest value for carcinogenic risk appears in sediment sample K2, mainly because of Cr. On the other hand, samples from the chlor–alkali plant (D) and the surface samples from the electrolysis facility (EF) have the highest pollution indices (ERI, PLI, and R) and HI (total hazard index, the sum of element HQs from three different exposure routes for each sample (Figure 5a)). Hg is the main element responsible for those values.

A different situation was obtained for the non-industry samples. Between all variables, except for ERI, the correlations are good and significant (Table 4b). PCA analysis of HI and cancer risk with pollution indices in the non-industrial area (Figure 5b) showed the highest values of non-carcinogenic risk and pollution indices in the samples from Vojlovica (V) and Messer (M). The lowest values of HI and pollution indices have samples from the rural sampling points in Starčevo (S). The highest ERI values, mostly contributed by Hg, were obtained in a surface sample from Pančevo city (PZ) and the Messer locality (M). The highest contribution to the HI of the residential area has Co, Mn, and As (Figure 4), and for carcinogenic risk, Cr and As.

## 3. Experimental

### 3.1. Description of the Study Area and Samples

We collected thirty-six soil samples from 0–100 cm depth at 9 sampling locations and five sediment samples in the area of the Pančevo Petrochemical Industry and its vicinity. Pančevo (44°52’15” North, 20°38’25” East) is an industrial town located in Vojvodina, the northern province of the Republic of Serbia [30,31].

The analyzed soil and sediment samples are from within and around the Petrochemical Industry of Pančevo, Serbia, and from the wastewater channel. This channel was built in 1962 to collect the wastewater discharges from the industrial complex of Pančevo city (Figure 2). The industrial complex consists of a chemical fertilizer factory, a petrochemical factory, and an Oil Refinery [32,33]. Within the petrochemical factory is a wastewater treatment plant designed to accept and process wastewater from all Petrochemical production plants and from the Oil Refinery and its effluent waste is discharged to the wastewater channel. The channel is an artificial channel without natural flow and carries the wastewaters directly to the Danube.

Samples were collected at each sampling site as composite samples consisting of four soil and sediment sub-samples. Sampling sites within the Petrochemical complex were: the Chlor–Alkali Plant (D), the Electrolysis Facility (EF), along the road in the Petrochemical Industry (P), the area for Mercury Disposal (ZD), along the road around the Petrochemical Industry (PP), and sampling sites in the vicinity Messer Industry (M), Vojlovica (V), Starčevo (S), Pančevo City (PZ), and wastewater channel (K) (Figure 2). The number beside the abbreviation of the sampling location indicates the sampling depth. Soil samples were sampled from these depths: 0–5 cm; 10–15 cm; 20–25 cm; 45–50 cm, and 90–100 cm. Within some sampling points, because of the ground hardness, depths of 45–50 and 90–100 cm were not sampled. The soil and sediment samples were packed in pouches and stored at 4 °C to prevent changes in chemical composition prior to analysis.

### 3.2. Microwave Digestion

Each sample was air-dried, sieved through 2 mm stainless steel sieves, and ground to a fine powder. We determined the hygroscopic moisture by drying samples at 105 °C until they were a constant dry weight. The analyzed samples were digested in a Milestone Ethos 1 microwave oven in sealed PTFE (polytetrafluoroethylene) vessels using 3 mL HNO_3_ (p.a., ≥65%) and 9 mL HCl (p.a., ≥37) for 500 mg ± 1 mg of the sample to obtain the pseudo-total content of the elements. The temperature was raised to 165 °C in 10 min, then to 175 °C in 3 min, and then kept constant for 10 min (the maximum power was 1200 W) [31,34]. The cooling solutions were transferred to 100 mL volumetric flasks and diluted to volume with deionized water.

### 3.3. Elemental Analysis

In the extracts, after microwave digestion of the samples, the concentration of Ba, Cd, Co, Cu, Cr, Mn, Ni, Pb, V, Zn, As, Hg, Se was determined using inductively coupled plasma–optical emission spectrometer (ICP/OES). External standard solutions were prepared from 1000 mg L^−1^ stock metal solutions (Multi-Element Plasma Standard Solution 4, Specpure (Alfa Aesar GmbH & Co KG, Germany)). The acid matrix baseline correction wavelengths for each metal were selected by comparing the observed signal intensities for the acid blank, the standard, and the sediment extract solution. We subtracted the blank intensity from both the standard and the sample intensities. A multi-element standard stock solution and blanks were prepared in the same matrix as extracting reagents to minimize matrix effects.

### 3.4. Quality of Measurements and Assurance

Quality assurance was done with BCR 143R certificate material for elements, after determining the pseudo-total content; good readability was achieved. Trueness was determined by comparing the measured concentration with the certificate value and is expressed as a percentage. The trueness goes over 100% in the case of Hg and Cr (114% and 113%, respectively) and for other elements, accuracies are: for Cd 94%, for Co 88%, for Cu 82%, for Mn 98%, for Ni 95%, for Pb 95%, and for Zn 85%. We replicated every 10th sample and precision calculated, as a percentage, using the standard deviation, and divided by the mean of the replicated measurements; the results were considered to be within ≤10% for all analyzed elements.

### 3.5. Data Processing

Calculation and statistical analyses were performed using Excel and OriginPro 9.0. The principal component (PCA) and correlation analysis were used to determine the distribution of elements and health risk criteria among the analyzed samples. For the risk assessment and calculations of non-carcinogenic and carcinogenic risks, we used equations that are present in the RAIS site (Risk Assessment Information System) [35] according to the guidelines and Exposure Factors Handbook of USE EPA (United States Environmental Protection Agency) [36,37,38,39,40].

#### 3.5.1. Environmental Assessment of Soil and Sediment Contamination

##### Enrichment Factor

This enrichment factor (EnF) is applied to differentiate between the anthropogenic and geochemical content of elements. It is calculated by using Equation (1):

(1) $$EnF={\left(\frac{C_{n}}{C_{ref}}\right)}_{sample}/{\left(\frac{C_{n}}{C_{ref}}\right)}_{background}$$

where C_n_ represents the element content in a sample and the element background content in the background sample, while C_ref_ is the content of the reference element within the analyzed and background samples in this study [41]. Al was adopted as a reference element in this study [2,42,43]. The EnF values are interpreted as the levels of element pollution suggested by Chen et al. [44]. EnF < 1 indicates no enrichment, EnF = 1–3 indicates minor enrichment, EnF = 3–5 indicates moderate enrichment, EnF = 5–10 indicates moderately severe enrichment, EnF = 10–25 indicates severe enrichment, EnF = 25–50 indicates very severe enrichment, and EnF > 50 indicates extremely severe enrichment.

##### Ecological Risk Index

Ecological risk index (ERI), which is also called potential ecological risk index in some articles [42], is critical for measuring both risk factor and element content in soil and sediment samples. The possible ecological risk index can be determined using the following formula:

(2) $$ERI=\sum \left(T_{ri}\cdot C_{fi}\right)$$

where Tr_i_ is the biological toxic factor. For some of the toxic and trace elements these factors are Cr = 2, Cd = 30, Cu = 5, Pb = 5, Ni = 5, Zn = 1, As = 10, and Hg = 40, as suggested by Hakanson [45]. C_fi_ is a single contamination factor, which is evaluated by dividing the content of the element in samples with its background value. ERI represents the qualitative scale of the pollution intensity of samples, which can be classified as follows: ERI < 150—low ecological risk; 150 < ERI < 300—moderate ecological risk; 300 < ERI < 600—considerable risk; and ERI > 600—very high ecological risk. This factor represents the sensitivity of the biological community to the toxic substances and illustrates the potential ecological risk caused by the overall contamination [42,46,47].

##### Total Enrichment Factor (R)

Beside the previously mentioned ecological indices, there is a so-called “total enrichment factor”, R [48,49], which represents the average of all metal or metalloid r values by this equation:

(3) R=(∑r)/n

while r represents the ratio between the difference of the concentration of an element in the sample and its concentration in the background, the sample and element concentration in the background sample are:

(4) $$r=\left(C_{n}-C_{background}\right)/C_{background}$$

where n is a number of analyzed elements.

A total enrichment factor (R) value exceeding 1.5 indicates high pollution, while values between 1.5 and 1 imply to moderate pollution, and lower values, and values below 0, indicate the unpolluted area of investigation.

##### Pollution Load Index (PLI)

The PLI index provides an assessment of the level of trace element pollution for the entire sampling site and represents the *n*th root of the product of thecontamination factor, which represents the ratio of element concentration to the background concentration of the corresponding element [1,41,42]. By using this equation, PLI was calculated as

(5) $$PLI=\sqrt[{n}]{(C_{fi1}\cdot C_{fi2}\cdot C_{fi3}\cdot \cdots C_{fin}}$$

According to the PLI, a sampling site can be classified into four groups: (i) nonpolluted (if PLI < 1), (ii) moderately polluted (if 1 ≤ PLI < 2), (iii) heavily polluted (2 ≤ PLI < 3), and (iv) extremely polluted (PLI ≥ 3) [50].

#### 3.5.2. Health Risk Assessment

A model of health risk assessment (HRA) was developed to establish potential health risks posed to humans and caused by contaminants in different environmental compartments [1,6,9,27,51,52]. Since part of the investigated area is located within the industrial zone of the petrochemical industry, adequate equations for composite workers were used [35,36]. The other part of the investigated samples is located within the non-industrial area around the petrochemical industry, so equitation’s adjustable equation for residential soil exposure was also used [35,36,37,53,54]. The chronic daily intake (CDI) was calculated separately for each element through individual exposures. The calculated CDI was subsequently divided with the corresponding reference dose (RfD) or reference concentration (RfC) [36,55] to yield the hazard quotient (HQ). The calculated CDI values were multiplied with the cancer slope factors (CSF) [55,56] for oral and dermal exposure, while for inhalation exposure [57], the values were multiply with unit risk factors (IUR) [36,58], to obtain the cancer risk.

The equations for the calculation of health risk assessment are the following:

(6) $$CDI_{oral}=\frac{C_{element}\cdot IR\cdot EF\cdot ED\cdot RBA}{AT\cdot BW}$$

(7) $$CDI_{dermal}=\frac{C_{element}\cdot EF\cdot ED\cdot SA\cdot AF\cdot ABS}{AT\cdot BW}$$

(8) $$CDI_{inh}=\frac{C_{element}\cdot EF\cdot ED\cdot ET\cdot \left(\frac{1}{PEF}\right)}{AT}$$

where C_element_ is the content of elements in samples (mg kg^−1^); IR is the ingestion rate (100 mg day^−1^); EF is exposure frequency (250 days year^−1^); ED is exposure duration (25 years); RBA is a relative bioavailability factor; for As this value is 0.6, while for the rest of elements it is 1 (that is, 100%) [59]; AT is the average time in days (365 days year^−1^·ED (years)), and for cancer risk, the AT value was 25,500 days (365 days year^−1^·70 years); BW is body weight (80 kg); SA is surface area of the skin in contact with soil (3527 cm^2^ day^−1^); AF is skin adherence factor for soil (0.12 mg cm^−2^); ABS is the dermal absorption factor (0.01 for all elements, except for Cd, which is 0.001, and As, which is 0.03) [60]; ET is exposure time (8 h days^−1^; and PEF is a particulate emission factor (1.36·10^9^ m^3^ kg^−1^) [37,53,54]. PEF represents pollutants that are adsorbed via respirable particulate matter (PM10) and relates to the concentration of contaminants in soil with the concentration of respirable particles in the air, due to fugitive dust emissions [54,57].

The above variables in equations are for the composite workers while the equations are slightly different for the residential exposure.

The equations for oral exposure are

(9) $$CDI_{oral}=\frac{C_{element}\cdot IR_{adj}\cdot RBA}{AT}$$

(10) $$IR_{adj}=\left(\frac{ED_{child}\cdot EF_{child}\cdot IR_{child}}{BW_{child}}+\frac{\left(ED_{adult}-ED_{child}\right)\cdot EF_{adult}\cdot IR_{adult}}{BW_{adult}}\right)$$

We calculated IR_adj_ (adjustable) for children and adults exposed to the potentially toxic elements from the soil by combining the exposure parameters and ingestion rates. The exposure duration for a child, according to US EPA [39], is 6 years but 26 years for adults. Exposure frequency for children and adults is 350 days in a year, while the ingestion rate for a child is 200 mg per day and 100 mg per day for an adult. Body weight is also different. For a child, body weight is 15 kg, while for an adult it is 80 kg. In the above CDI_oral_ exposure in the denominator, the ED is 26 years for non-carcinogenic exposure, while for carcinogenic exposure in residential areas, the ED is 70 years.

For adjustable residential dermal exposure the equations are

(11) $$CDI_{dermal}=\frac{C_{element}\cdot D_{adj}\cdot ABS}{AT}$$

(12) $$D_{adj}=\left(\frac{ED_{child}\cdot EF\cdot SA_{child}\cdot AF_{child}}{BW_{child}}+\frac{\left(ED_{adult}-ED_{child}\right)\cdot EF\cdot SA_{adult}\cdot AF_{adult}}{BW_{adult}}\right)$$

where the ED and EF values are the same as they are in IR_adj_, while in D_adj_ (dermal adjustable), the SA (surface area) for a child is 2373 cm^2^ per day and 6032 cm^2^ per day for an adult. The AF (adherence factor) [53] for a child is 0.2 mg per cm^2^ and 0.07 mg per cm^2^ for an adult. The body weights for children and adults are 15 and 80 kg, respectively [53]. In the above CDI_dermal_ exposure in the denominator, the ED is 26 years for non-carcinogenic exposure, while for carcinogenic exposure in residential area, the ED in the denominator is 70 years.

Within the calculation of dermal non-carcinogenic (hazardous) and carcinogenic exposure, the GIABS values (Gastrointestinal Absorption Factor) of elements were used to determine the RfD_dermal_ (RfD_oral_·GIABS) and CFS_dermal_ (CSF_oral_/GIABS). For calculations of non-carcinogenic (RfD and RfC) and carcinogenic risks (CSF and IUR), elemental toxicological values were used and are presented on the RAIS website [36] (Table S4), while in the case of Pb, we used an RfD value of 3.5·10^−3^ mg kg^−1^ day^−1^ [55], and for cancer slope and unit risk, we used RfD values of 8.5·10^−3^ kg day mg^−1^ [56] and 1.2·10^−5^ m^3^ µg^−1^, respectively [58], while the GIABS value was 1 [36].

## 4. Conclusions

Within the petrochemical complex, the most polluting element is Hg, especially in the chlor–alkali plant where the EnF values were above 6000. Besides this location, samples from the electrolysis factory, mercury disposal area, and sediments from the wastewater channel show extremely severe enrichment in quantities of Hg. Besides mercury, Cu, Pb, Zn, Cd, and Cr are pollutants of samples from the industrial area and Hg, Cr, and Cd in sediment samples are found in the wastewater channel. In samples from the non-industrial area, samples located northeastern from the industry have higher Hg concentrations with elevated ERI values, while on the other side, Cr is the element responsible for higher carcinogenic risks in both sets of samples. Elements that mainly contribute to higher HQ values in the non-industry area are Mn, Co, and V. Within this work, the worst-case scenario was considered, and the values of carcinogenic risks were above 1·10^−4^, which is the upper limit of acceptable risk. Beside Cr, As is an element that contributes to the elevated values of carcinogenic risk, especially within the non-industrial area, since these samples have higher concentrations of this metalloid.

Although the petrochemical complex, and the chlor–alkali plant within, were partly destroyed in 1999, for the area outside of the complex, Hg is not a relevant pollution source, while the role of Cr and As, and Co, Mn, and V remains unclear. On the other hand, when occupational exposure is considered, the detected Hg concentrations represent a significant source of pollution and a threat to the health of workers.

## Figures and Tables

**Table 1:** Mean, median, minimum, and maximum values, with standard deviations, the geometric mean of analyzed metals, with background values and maximum allowable contents.

	Al	Ba	Cd	Co	Cr	Cu	Mn	Ni	Pb	V	Zn	As	Hg	Se
Mean value within industrial area (mg kg^−1^)	21,251	183	0.24	7.1	87	110	430	37	56	43	202	6.3	35.6	0.19
Std. Deviation within industrial area (mg kg^−1^)	15,207	160	0.29	3.1	198	300	185	13	87	28	298	2.9	69.2	0.27
Mean value from non-industrial area (mg kg^−1^)	41,333	245	0.06	11.4	61	30	700	48	36	76	128	11.0	0.6	0.14
Std. Deviation from non-industrial area (mg kg^−1^)	5419	38	0.04	1.2	10	9	83	12	15	10	66	1.7	0.3	0.06
Maximum for both sets of samples (mg kg^−1^)	54,576	688	1.23	14.1	1032	1108	840	75	337.0	97	1140	15.3	237.5	1.33
Coefficient of variation within industrial area (%)	72	87	121	44	228	273	43	35	155	65	148	46	194	142
Coefficient of variation within non-industrial area (%)	13	16	67	11	16	30	12	25	42	14	52	15	50	43
Background values from the study (mg kg^−1^)	45,732	283	0.08	12.1	71	26	691	60	41.7	83	150	12.9	0.3	0.16
Background values from Turekian and Wedepohl [24] (mg kg^−1^)	80,000	580	0.3	19	90	45	850	68	20	130	95	13.0	0.4	0.6
Maximum allowable contents of elements in Serbia (mg kg^−1^) [25]	-	-	3	-	100	100	-	50	100	-	300	25	2	-
>TEC^a^ within industrial area (%)	-	-	48	-	48	16	-	92	32	-	32	8	88	-
>PEC^b^ within industrial area (%)	-	-	0	-	4	12	-	16	12	-	12	0	76	-
>TEC from non-industrial area (%)	-	-	0	-	94	38	-	100	56	-	44	69	100	-
>PEC from non-industrial area (%)	-	-	0	-	0	0	-	44	0	-	0	0	12	-

^a^ TEC: threshold effect concentration [12]. ^b^ PEC: probable effect concentration [12].

**Table 2:** Enrichment factors (EnF) of analyzed elements.

Sampling Point	Ba EnF	Cd EnF	Co EnF	Cr EnF	Cu EnF	Mn EnF	Ni EnF	Pb EnF	V EnF	Zn EnF	As EnF	Hg EnF	Se EnF
D	19.1 ± 4.0	57.8 ± 30.1	2.26 ± 0.66	6.43 ± 0.91	276 ± 162	2.59 ± 0.32	2.88 ± 1.18	67.7 ± 19.8	1.65 ± 0.41	64.4 ± 18.2	1.18 ± 0.30	6066 ± 1173	23.7 ± 5.5
EF	1.00 ± 0.16	11.3 ± 12.9	2.38 ± 0.16	2.95 ± 0.95	2.19 ± 2.27	2.33 ± 0.01	3.26 ± 0.46	2.03 ± 1.39	1.29 ± 0.28	3.00 ± 1.87	2.26 ± 0.32	896 ± 1028	4.58 ± 4.89
P	0.88 ± 0.03	0.71 ± 0.39	1.03 ± 0.05	0.95 ± 0.03	0.93 ± 0.19	1.14 ± 0.14	0.85 ± 0.07	0.75 ± 0.33	1.02 ± 0.04	0.58 ± 0.10	0.87 ± 0.03	7.85 ± 1.76	0.81 ± 0.08
ZD	0.87 ± 0.11	9.14 ± 4.89	2.12 ± 0.67	2.18 ± 0.61	1.62 ± 1.11	2.12 ± 0.72	2.59 ± 0.97	1.72 ± 0.87	1.10 ± 0.05	1.87 ± 0.40	2.22 ± 1.06	82.4 ± 104.4	0.77 ± 0.21
PP	0.88 ± 0.13	1.48 ± 0.86	1.17 ± 0.08	0.98 ± 0.04	0.84 ± 0.05	1.23 ± 0.05	0.89 ± 0.04	0.84 ± 0.56	1.03 ± 0.02	0.75 ± 0.02	0.80 ± 0.06	1.42 ± 0.80	0.81 ± 0.10
M	0.94 ± 0.05	0.92 ± 0.28	0.96 ± 0.02	0.90 ± 0.01	1.58 ± 0.32	1.09 ± 0.05	0.82 ± 0.04	0.89 ± 0.76	0.99 ± 0.02	0.72 ± 0.11	0.87 ± 0.04	2.62 ± 0.98	0.81 ± 0.26
V	1.00 ± 0.04	0.85 ± 0.38	1.09 ± 0.05	1.05 ± 0.05	1.32 ± 0.11	1.16 ± 0.03	1.11 ± 0.11	1.07 ± 0.05	1.04 ± 0.01	1.04 ± 0.14	1.02 ± 0.12	1.60 ± 0.44	1.41 ± 0.11
S	0.92 ± 0.02	1.01 ± 0.90	1.18 ± 0.02	0.91 ± 0.02	1.02 ± 0.11	1.14 ± 0.01	0.78 ± 0.01	0.86 ± 0.07	1.01 ± 0.02	1.29 ± 1.24	1.01 ± 0.03	1.81 ± 0.78	0.61 ± 0.25
PZ	0.94 ± 0.02	0.56 ± 0.72	0.96 ± 0.02	0.90 ± 0.02	1.25 ± 0.06	1.14 ± 0.02	0.75 ± 0.01	1.07 ± 0.03	1.00 ± 0.01	0.76 ± 0.09	0.88 ± 0.06	3.24 ± 0.72	0.92 ± 0.14
K	1.02 ± 0.08	14.2 ± 7.4	1.24 ± 0.36	13.6 ± 27.4	1.83 ± 0.83	1.48 ± 0.76	1.85 ± 0.98	2.19 ± 0.63	1.41 ± 0.38	2.76 ± 1.64	1.07 ± 0.41	81.8 ± 98.2	5.26 ± 4.90

**Table 3:** Ecological risk index, pollution load index, and total enrichment factor of analyzed sampling locations. ERI, ecological risk index; PLI, pollution load index; R, enrichment factor.

Sampling Point	ERI	PLI	R
D	24,740 ± 4690	1.9 ± 0.7	50.1 ± 10.3
EF	7416 ± 8732	0.75 ± 0.37	13.7 ± 17.0
P	335 ± 46	0.95 ± 0.20	0.33 ± 0.17
ZD	1238 ± 1499	0.59 ± 0.28	1.78 ± 3.00
PP	62 ± 19	0.45 ± 0.10	-0.50 ± 0.06
M	163 ± 34	1.00 ± 0.10	0.10 ± 0.09
V	115 ± 11	1.06 ± 0.05	0.08 ± 0.03
S	96 ± 36	0.71 ± 0.12	-0.22 ± 0.15
PZ	152 ± 49	0.84 ± 0.14	-0.04 ± 0.14
K	1307 ± 995	1.21 ± 0.57	2.57 ± 2.52

**Table 4:** Spearman correlations of pollution indices and health risk criteria within (**a**) the industrial area and (**b**) the non-industrial area (* for *p* < 0.050).

**(a)**	**PLI**	**R**	**ERI**	**HI**	**Cancer Risk**
PLI	1				
R	0.805 *	1			
ERI	0.748 *	0.987 *	1		
HI	0.916 *	0.852 *	0.800 *	1	
cancer risk	0.547	0.272	0.233	0.552 *	1
**(b)**	**PLI**	**R**	**ERI**	**HI**	**Cancer Risk**
PLI	1				
R	0.950 *	1			
ERI	0.446	0.636 *	1		
HI	0.932 *	0.846 *	0.254	1	
cancer risk	0.914 *	0.811 *	0.243	0.979 *	1

## Supplementary Materials

The following are available online, Table S1: Pseudo-total content of analyzed elements (mg kg^−1^), in samples from (a) petrochemical industry and (b) surroundings, Table S2: Pollution indices and HRA of samples from industrial area, Table S3: Pollution indices and HRA of samples from non-industrial area, Table S4: Toxicological values of PTE.
