# Supplementary material for: Pollution and Health Risk Assessments of Potentially Toxic Elements in Soil and Sediment Samples in a Petrochemical Industry and Surrounding Area
Source: Molecules. 2019 Jun 6;24(11):2139. doi: 10.3390/molecules24112139 (PMC6600396; doi:10.3390/molecules24112139)
Supplement: Supplementary file 1 [file molecules-24-02139-s001.pdf]

Table S1 . Pseudo-total content of analyzed elements (mg kg^{-1}) in samples from a) petrochemical industry and b) surroundings

a)	Al	Ba	Cd	Co	Cr	Cu	Mn	Ni	Pb	V	Zn	As	Hg	Se
D 5	5950	688	0.96	4.3	68	1090	248	32	337	23	1140	2.08	202.91	0.36
D 15	4764	449	0.37	1.9	41	1108	160	13	222.6	12	779	1.14	237.48	0.45
D 25	3426	493	0.22	2.3	34	187	145	11	280	9	953	1.38	162.25	0.32
EF 5	9513	61	0.43	5.7	34	26	336	39	29.1	28	161	5.11	142.77	0.34
EF 15	8273	59	0.10	5.6	52	4	291	41	4.4	17	55	5.92	23.14	0.06
EF 25	9762	50	0.02	6.0	38	5	341	37	19.1	20	58	6.44	15.59	0.05
P5	37588	195	0.04	10.4	58	25	742	44	28.8	73	80	9.18	2.57	0.09
P15	36576	199	0.01	10.4	54	21	681	45	6.4	69	76	9.35	2.65	0.10
P25	37766	216	0.04	10.8	57	21	665	43	36.2	70	82	9.36	1.81	0.12
P50	54576	308	0.10	14.1	78	22	814	56	41.7	97	83	12.68	2.59	0.16
P100	50572	271	0.10	13.3	72	22	782	51	39.3	90	80	12.65	2.37	0.15
ZD 5	14929	76	0.37	6.8	34	27	394	41	23.4	30	112	6.64	24.9	0.04
ZD 15	17907	114	0.29	7.3	68	24	392	43	5.3	34	101	6.78	19.92	0.05
ZD 25	16590	93	0.06	7.2	43	9	406	38	24.8	32	68	6.75	2.29	0.06
ZD 50	6495	30	0.08	5.0	24	4	275	32	14.6	13	43	6.40	0.15	0.01
ZD 100	6958	36	0.16	5.1	32	2	315	32	15.5	15	47	6.40	0.23	0.02
PP 5	25562	169	0.10	7.3	39	13	456	30	21.8	49	62	6.29	0.11	0.07
PP 15	22492	116	0.04	7.1	35	10	409	27	0.1	42	54	5.07	0.30	0.07
PP 25	22771	113	0.02	7.0	33	11	429	25	22.4	42	56	4.69	0.15	0.07
PP 50	20912	106	0.08	7.0	33	10	412	25	24.4	38	54	4.57	0.35	0.05
K1	19541	135	0.45	5.6	46	16	259	35	32.4	43	130	4.75	1.63	0.12
K2	10610	68	0.33	4.8	1032	16	339	48	29.1	37	167	4.91	19.19	0.28
K3	48041	270	0.31	12.0	76	29	526	51	60.0	90	152	8.11	4.77	0.36
K4	29541	194	1.23	7.3	54	47	560	70	68.6	91	405	7.80	14.68	1.33
K5	10156	62	0.22	4.1	28	7	377	24	20.3	22	61	3.69	4.70	0.07
Average	21251	183	0.25	7.1	87	110	430	37	56.3	43	202	6.33	35.58	0.19

b)	Al	Ba	Cd	Co	Cr	Cu	Mn	Ni	Pb	V	Zn	As	Hg	Se
M 5	44173	260	0.08	11.4	62	46	756	50	79.6	81	114	11.17	0.58	0.14
M 15	44844	278	0.04	11.2	62	40	743	48	13.4	81	115	11.10	0.78	0.16
M 25	50164	292	0.10	12.6	70	51	840	55	19.0	89	123	12.50	1.02	0.16
M 100	46311	253	0.08	11.9	65	30	707	47	35.4	81	83	10.57	1.15	0.07
V 5	43763	279	0.04	12	70	36	773	61	44.1	83	179	11.48	0.46	0.21
V 15	43489	283	0.10	12.2	71	34	741	63	44.6	82	145	11.85	0.43	0.23
V 25	42196	254	0.04	12.5	66	29	758	56	41.1	78	127	11.77	0.69	0.22
V 50	45116	272	0.08	13.7	79	32	802	75	41.2	86	145	15.32	0.43	0.20
V 100	45732	283	0.08	12.1	71	26	691	60	41.7	83	150	12.87	0.33	0.16
S 5	33738	187	0.10	10.4	47	21	582	35	24.1	63	348	9.19	0.68	0.06
S 15	32558	186	0.01	10.1	45	20	550	34	25.6	61	80	9.33	0.25	0.07
S 25	30582	173	0.01	9.6	43	18	531	31	23.4	57	67	8.70	0.29	0.04
S 50	39124	230	0.02	12.4	57	19	677	40	33.7	70	76	11.59	0.58	0.13
PZ 5	41253	234	0.10	10.2	57	29	714	41	41.1	75	116	9.41	1.21	0.15
PZ 15	41096	239	0.01	10.5	56	28	712	40	38.8	74	99	10.40	0.87	0.14
PZ 25	37186	221	0.01	9.6	53	28	628	36	36.4	67	84	9.62	0.73	0.10

Average	41333	245	0.06	11.4	61	30	700	48	36.5	76	128	11.05	0.66	0.14

Table S2. Pollution indices and HRA of samples from industrial area

	PLI	R	ERI	HI	Cancer Risk
D 5	2.64	52.22	25219	1.15	3.47E-05
D 15	1.70	59.25	29172	1.23	2.09E-05
D 25	1.37	38.98	19828	0.88	1.78E-05
EF 5	1.17	33.30	17484	0.76	1.91E-05
EF 15	0.55	4.81	2853	0.21	2.81E-05
EF25	0.52	2.99	1910	0.18	2.16E-05
P5	0.88	0.29	348	0.21	3.27E-05
P15	0.69	0.22	343	0.20	3.08E-05
P25	0.88	0.13	256	0.21	3.23E-05
P50	1.17	0.55	378	0.27	4.41E-05
P100	1.11	0.45	350	0.25	4.12E-05
ZD 5	0.88	5.62	3175	0.23	1.98E-05
ZD 15	0.84	4.40	2540	0.24	3.62E-05
ZD 25	0.61	0.01	315	0.14	2.42E-05
ZD 50	0.29	-0.63	59	0.08	1.49E-05
ZD 100	0.34	-0.52	99	0.10	1.87E-05
PP 5	0.54	-0.43	65	0.14	2.20E-05
PP 15	0.32	-0.54	61	0.12	1.94E-05
PP 25	0.44	-0.55	38	0.12	1.83E-05
PP 50	0.50	-0.47	84	0.12	1.83E-05
K1	0.80	0.31	382	0.13	2.46E-05
K2	1.25	5.44	2494	0.97	4.98E-04
K3	1.42	1.35	721	0.24	4.09E-05
K4	2.01	5.16	2274	0.27	3.02E-05
K5	0.55	0.62	662	0.11	1.54E-05
Mean	0.94	8.52	4444	0.34	4.50E-05
StDev	0.57	17.31	8499	0.35	9.48E-05
Min	0.29	-0.63	38	0.08	1.49E-05
Max	2.64	59.25	29172	1.23	4.98E-04

Table S3. Pollution indices and HRA of samples from non-industrial area

	PLI	R	ERI	HI	Cancer Risk
M 5	1.07	0.12	134	0.92	1.12E-04
M 15	0.90	0.00	134	0.84	1.11E-04
M 25	1.10	0.22	190	0.95	1.26E-04
M 100	0.94	0.07	194	0.87	1.15E-04
V 5	1.03	0.06	100	0.93	1.25E-04
V 15	1.10	0.11	119	0.92	1.27E-04
V 25	1.00	0.05	126	0.91	1.19E-04
V 50	1.12	0.12	115	1.02	1.44E-04
S 5	0.84	-0.04	141	0.70	8.58E-05
S 15	0.58	-0.37	53	0.66	8.29E-05
S 25	0.63	-0.33	90	0.63	7.89E-05
S 50	0.77	-0.16	100	0.82	1.05E-04
PZ 5	1.00	0.12	208	0.81	1.02E-04
PZ 15	0.80	-0.06	133	0.81	1.01E-04
PZ 25	0.72	-0.16	115	0.74	9.57E-05
Mean	0.91	-0.02	130	0.84	1.09E-04
StDev	0.17	0.17	40	0.11	1.76E-05
Min	0.58	-0.37	53	0.63	7.89E-05
Max	1.12	0.22	208	1.02	1.44E-04

Table S4. Toxicological values of PTE

	RfD (mg $kg^{-1} \cdot day^{-1}$)	RfC (mg m^{-3})	CSF (kg·day mg^{-1})	IUR (m^3 μg^{-1})	GIABS	ABS
Ba	2.00E-01	5.00E-04			7.00E-02	1.00E-02
Cd	1.00E-03	1.00E-05		1.80E-03	2.50E-02	1.00E-03
Co	3.00E-04	6.00E-06		9.00E-03	1.00E+00	1.00E-02
Cu	4.00E-02				1.00E+00	1.00E-02
Cr	3.00E-03	1.00E-04	5.00E-01	1.00E-04	2.50E-02	1.00E-02
Mn	2.40E-02	5.00E-05			4.00E-02	1.00E-02
Ni	1.10E-02	1.40E-05		2.40E-04	4.00E-02	1.00E-02
Pb	3.50E-03		8.50E-03	1.20E-05	1.00E+00	1.00E-02
V	5.00E-03	1.00E-04			2.60E-02	1.00E-02
Zn	3.00E-01				1.00E+00	1.00E-02
Hg	3.00E-04	3.00E-04			7.00E-02	1.00E-02
As	3.00E-04	1.50E-05	1.50E+00	4.30E-03	1.00E+00	3.00E-02
Se	5.00E-03	2.00E-02			1.00E+00	1.00E-02
